# The Epigenetic Reader Protein SP140 Regulates Dendritic Cell Activation, Maturation and Tolerogenic Potential

**DOI:** 10.3390/cimb45050269

**Published:** 2023-05-11

**Authors:** Mohammed Ghiboub, Matthew Bell, Dovile Sinkeviciute, Rab K. Prinjha, Menno P. J. de Winther, Nicola R. Harker, David F. Tough, Wouter J. de Jonge

**Affiliations:** 1 Tytgat Institute for Liver and Intestinal Research, Amsterdam Gastroenterology, Endocrinology Metabolism, Amsterdam University Medical Centers, Location AMC, University of Amsterdam, 1105 BK Amsterdam, The Netherlands; m.ghiboub@amsterdamumc.nl; 2 Immunology Research Unit, Medicines Research Centre, GlaxoSmithKline, Stevenage SG1 2NY, UK; matthew.x.bell@gsk.com (M.B.); dovile.sinkev@gmail.com (D.S.); rabinder.prinjha@gsk.com (R.K.P.); nicola.r.harker@gsk.com (N.R.H.); 3 Department of Medical Biochemistry, Amsterdam University Medical Centers, University of Amsterdam, 1105 AZ Amsterdam, The Netherlands; m.dewinther@amsterdamumc.nl; 4 Department of Medicine, Institute for Cardiovascular Prevention (IPEK), 80336 Munich, Germany; 5 Department of Surgery, University of Bonn, 53127 Bonn, Germany

**Keywords:** SP140, GSK761, dendritic cells, maturation, tolerogenic potential

## Abstract

SP140 is an epigenetic reader protein expressed predominantly in immune cells. GWAS studies have shown an association between *SP140* single nucleotide polymorphisms (SNPs) and diverse autoimmune and inflammatory diseases, suggesting a possible pathogenic role for SP140 in immune-mediated diseases. We previously demonstrated that treatment of human macrophages with the novel selective inhibitor of the SP140 protein (GSK761) reduced the expression of endotoxin-induced cytokines, implicating a role of SP140 in the function of inflammatory macrophages. In this study, we investigated the effects of GSK761 on in vitro human dendritic cell (DC) differentiation and maturation, assessing the expression of cytokines and co-stimulatory molecules and their capacity to stimulate T-cell activation and induce phenotypic changes. In DCs, lipopolysaccharide (LPS) stimulation induced an increase in SP140 expression and its recruitment to transcription start sites (TSS) of pro-inflammatory cytokine genes. Moreover, LPS-induced cytokines such as TNF, IL-6, and IL-1β were reduced in GSK761- or *SP140* siRNA- treated DCs. Although GSK761 did not significantly affect the expression of surface markers that define the differentiation of CD14^+^ monocytes into immature DCs (iDCs), subsequent maturation of iDCs to mature DCs was significantly inhibited. GSK761 strongly reduced expression of the maturation marker CD83, the co-stimulatory molecules CD80 and CD86, and the lipid-antigen presentation molecule CD1b. Finally, when the ability of DCs to stimulate recall T-cell responses by vaccine-specific T cells was assessed, T cells stimulated by GSK761-treated DCs showed reduced *TBX21* and *RORA* expression and increased *FOXP3* expression, indicating a preferential generation of regulatory T cells. Overall, this study suggests that SP140 inhibition enhances the tolerogenic properties of DCs, supporting the rationale of targeting SP140 in autoimmune and inflammatory diseases where DC-mediated inflammatory responses contribute to disease pathogenesis.

## 1. Introduction

Dendritic cells (DCs) are a heterogeneous group of functionally specialized antigen-presenting cells that play a pivotal role in linking innate and adaptive immunity [1]. Communication between DCs and T-cells is one of the critical aspects controlling immune response induction, which under homeostasis is tightly regulated, and, thereby, is essential for immunity and tolerance [2,3]. Such a process is tightly controlled by epigenetic mechanisms, especially post-translational modification of histone tails [4,5]. For instance, specific histone acetylases and deacetylases are implicated in DC activation and maturation and in their potential to activate T cells [6]. A failure of this regulation can result in the aberrant and prolonged inflammatory responses seen in autoimmune and inflammatory diseases [7,8].

Speckled 140 KDa (SP140) is a nuclear body protein predominantly expressed in immune cells [9,10]. The expression of SP140 is induced during microbial infections and in response to inflammatory stimuli [9,10]. It has been reported that SNPs in the *SP140* locus have been strongly linked to diverse autoimmune and inflammatory diseases such as multiple sclerosis (MS) [11] and Crohn’s disease (CD) [12]. In addition, our previous study demonstrated upregulated expression of SP140 in a range of inflammatory diseases such as CD, rheumatoid arthritis (RA), and systemic lupus erythematosus [10], implicating SP140 in immune-mediated pathogenesis. SP140 is an epigenetic reader protein harboring a bromodomain (Brd) [12,13]; Brd recognizes acetylated lysine residues on histone tails and regulates gene expression through mechanisms that modulate DNA accessibility to transcription factors (TFs) and the transcriptional machinery [14,15].

Due to the integral role of DCs in the induction of an adaptive immune response, and their potential to both prevent and stimulate autoreactivity [16], transcriptional programs mediating those processes are an interesting target opportunity for anti-inflammatory therapies. Previous in vitro and in vivo studies have demonstrated a key role of Brd-containing proteins (BCPs) in controlling DC functions, particularly in the context of inflammation [17]. For instance, multiple inhibitors targeting BRD2, BRD3, and BRD4 have been shown to impair DC maturation and enhance their tolerogenic properties, enabling their ability to stimulate the differentiation of naïve T cells into FOXP3 expressing functional Tregs [17,18,19]. In a recent study, we reported the development of the first small molecule SP140 inhibitor (GSK761), which shows a selective ability to bind to and inhibit the function of SP140 compared to other BCPs [10]. SP140 inhibition using GSK761 strongly suppressed macrophage-induced cytokines and co-stimulatory molecules (such as *IL12, IL1β*, and *CD80)* required for inflammatory T-cell activation and was associated with inhibition of SP140 binding to the regulatory regions of these genes [10]. In addition, the SP140 protein was found to bind active regions of multiple human leukocyte antigen (*HLA*) genes in inflammatory macrophages [10]. These data suggest that SP140 may have a role in regulating antigen presentation.

Here we investigated the effects of SP140 inhibition with GSK761 on DC function using in vitro monocyte-derived DCs. We demonstrate a marked effect of SP140 inhibition on DC maturation, reducing their capacity to secrete pro-inflammatory cytokines and T-cell activation and enhancing the potential of SP140-treated DCs to induce FOXP3-expressing T cells. These results highlight the potential for SP140 to target inflammatory and autoimmune disorders.

## 2. Results

### 2.1. LPS-Stimulation Induces an Increase in SP140 Expression in DCs as Well as Its Recruitment to TSS of Pro-Inflammatory Cytokine Genes

**Figure 1:**
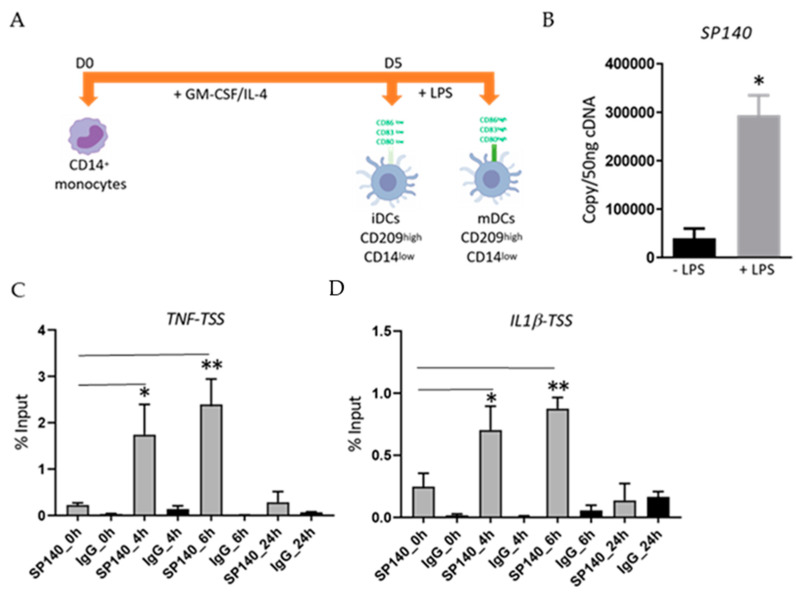
LPS-stimulation enhances SP140 protein recruitment to TSS of *TNF* and *IL1β.* (**A**) Protocol for an in vitro generation of DCs. Human primary CD14^+^ monocytes were incubated with 30 ng/mL GM-CSF and 20 ng/mL IL-4 for 5 days (**D**) to generate immature dendritic cells (iDCs). To generate mature DCs (mDCs), iDCs were washed with PBS and then stimulated with 100 ng/mL LPS. The illustration was created with BioRender.com. (**B**) *SP140* gene expression was measured by qPCR in naïve iDCs or after 4 h of 100 ng/mL LPS-stimulation, from 3 individual donors (*n* = 3). (**C**) ChIP-qPCR of SP140 protein occupancy at TSS of *TNF* (*n* = 4) and (**D**) *IL1β* (*n* = 3) in naïve iDCs or after 4, 6 or 24 h of 100 ng/mL LPS-stimulation, IgG was used as negative control. Statistical significance is indicated as follow: * *p* < 0.05 and ** *p* < 0.01. The error bars in a column graph represent standard deviation (SD).

To examine the expression of SP140 in DCs, primary human CD14^+^ monocytes were differentiated in vitro into immature DCs (iDCs) and then kept unstimulated or stimulated with LPS (Figure 1A). First, we verified the successful generation of DCs by assessing the expression of some specific surface markers by FACS as described in [20,21,22,23,24]. The cells were shown to be CD209^++^CD11c^++^CD14^low^, confirming their differentiation into iDCs (Supplementary Figure S1A,B). Low levels of *SP140* gene expression (Figure 1B) were detected in unstimulated iDCs. However, LPS stimulation significantly augmented *SP140* mRNA expression (Figure 1B). To assess whether SP140 associates with chromatin in DCs and how this might be regulated during inflammation, we conducted ChIP-qPCR experiments in unstimulated or LPS-stimulated (for 4, 6, and 24 h) iDCs targeting the transcription start site (TSS) of inflammatory cytokine genes; *TNF* and *IL1β*. The binding of SP140 to the TSS of *TNF* and *IL1β* genes was observed in unstimulated cells, and this was strongly increased following 4 and 6 h of LPS stimulation (Figure 1C,D). Notably, SP140 binding to those TSS after 24 h of LPS stimulation returned to baseline levels, suggesting the requirement of SP140 for pro-inflammatory cytokine gene expression early after endotoxin exposure. SP140 also binds to the TSS of *IL10*, but there was no increase in this binding after LPS stimulation (Supplementary Figure S2).

### 2.2. GSK761 Attenuates the Inflammatory Activation of DCs

**Figure 2:**
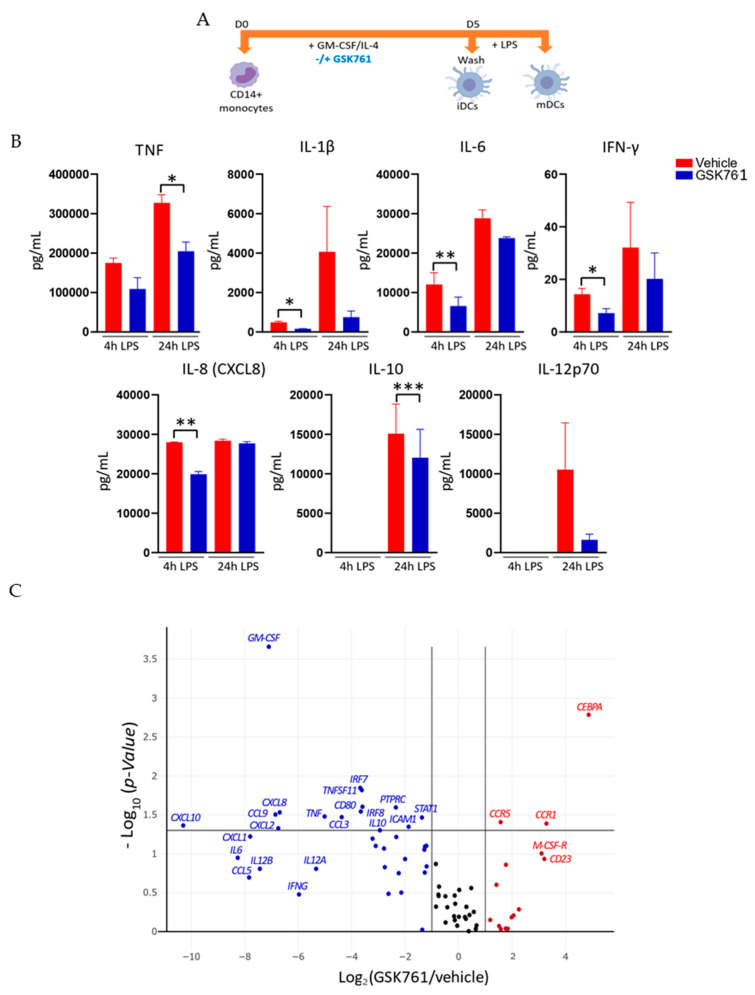
GSK761 reduces the inflammatory response of DCs. (**A**) Experimental set up for SP140 inhibition in DCs. Human primary CD14^+^ monocytes were incubated with 30 ng/mL GM-CSF and 20 ng/mL IL-4 for 5 days in presence of vehicle (0.1% DMSO) or 0.12 µM GSK761. The cells were then washed with PBS and stimulated for 4 or 24 h with 100 ng/mL LPS. The illustration was created with BioRender.com. (**B**) Protein level of cytokines was measured by MSD in the supernatant at both LPS-stimulation time points (4 or 24 h) (100 ng/mL), *n* = 4. (**C**) The impact of GSK761 on LPS-induced genes was assessed at 4 h poststimulation using RT2 Profiler™ PCR Array-Human Dendritic and Antigen Presenting Cell PCR Array (384-well 4 × 96), *n* = 4. Statistical significance is indicated as follow: * *p* < 0.05, ** *p* < 0.01, *** *p* < 0.001.

LPS-induced DCs maturation is typically associated with the activation of an inflammatory transcriptional program, including those required for T-cell activation [17]. In order to examine the requirement of SP140 in regulating the inflammatory response in DCs, the expression of the pro-inflammatory cytokines was examined in the vehicle (DMSO)- or GSK761-treated iDCs stimulated with LPS (to induce maturation to mDCs) (Figure 2A). We first tested whether GSK761 elicited any cytotoxicity in DCs using ATP-based analysis to determine the dose range used. DCs were incubated with 0.1% vehicle or with an increasing concentration of GSK761 prior to LPS stimulation. At concentrations ≤ 0.12 µM, GSK761 showed no cytotoxicity (Supplementary Figure S3A). These data are in line with our previous observation on human macrophages [10]. Similarly, in live/dead staining by FACS, DCs treated with 0.12 µM exhibited a high percentage of viability (Supplementary Figure S3B); thus, 0.12 µM GSK761 was used in subsequent experiments. At 4 or 24 h of LPS stimulation, GSK761-treatment led to reduced secretion of LPS-induced IL-6, TNF, IL-1β, IL-10, and IL-8 proteins (Figure 2B). In addition, SP140 inhibition reduced the expression of several other pro-inflammatory cytokine and chemokine genes, such as *GM-CSF*, *CCL3, CCL5, CCL9, CXCL10*, and *CCL1* (Figure 2B), and the transcription factor (TF) *STAT1* required for DC maturation [25]. The transcription of genes that typically mark iDCs, *CCR1* and *CCR5* [26], was significantly increased in GSK761-treated cells (Figure 2C). In addition, GSK761 inhibited gene expression of DC adhesion (*ICAM1*) and co-stimulatory molecules (*CD80*), which are required for inflammatory T-cell activation. To further validate the previous observations concerning the role of SP140 in regulating the expression of pro-inflammatory cytokines, we performed siRNA-mediated knock-down to reduce SP140 expression in DCs, achieving a significant reduction in *SP140* expression (Supplementary Figure S4A). SP140 knock-down yielded a significant decrease in LPS-induced TNF and IL-1β secretion (Supplementary Figure S4B).

To understand whether GSK761 directly affects the transcription of inflammatory cytokines and chemokines (that were observed in Figure 2) in myeloid cells by reducing SP140 binding at these genes, we re-analyzed our previous publicly available ChIP-sequencing data on SP140 binding in human inflammatory macrophages pretreated with GSK761 or DMSO. The data were obtained from the European Genome-phenome Archive: EGAS00001004460 [10]. By comparing peak signals resulting from DMSO- vs. GSK761-treated LPS-stimulated macrophages as described in [10], we observed that GSK761 strongly reduces the binding of SP140 to pro-inflammatory cytokine genes such as *IL1β, CXCL8* and *IL10* (Supplementary Figure S5A). Focusing on transcription start sites (TSS), the TSS plot illustrates the potential of GSK761 to inhibit the binding of SP140 at the TSS of genes encoding multiple cytokines and chemokines (we focused on the differentially expressed genes observed in Figure 2), including *TNF* and *CCL5* (Supplementary Figure S5B).

### 2.3. SP140 Is Critical for DCs Maturation

**Figure 3:**
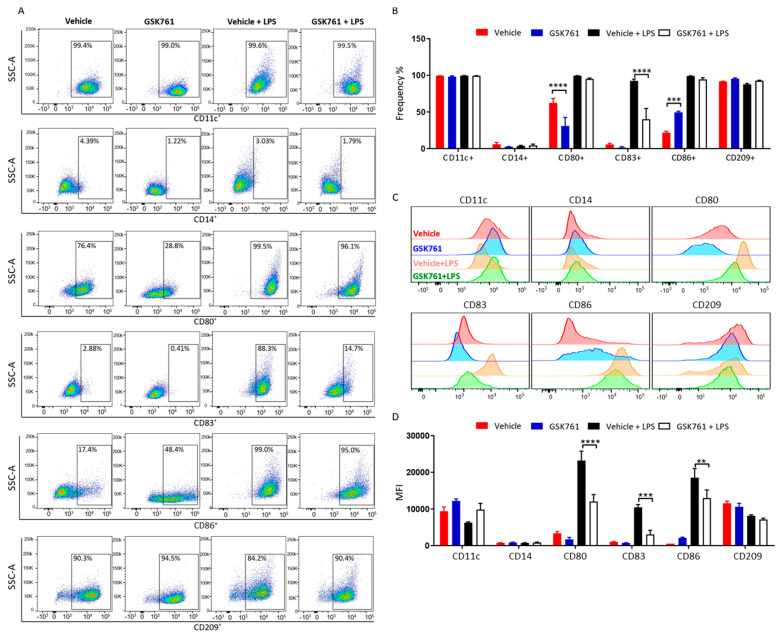
GSK761 impairs DCs maturation. Human primary CD14^+^ monocytes were incubated with 30 ng/mL GM-CSF and 20 ng/mL IL-4 for 5 days in presence of vehicle (0.1% DMSO) or 0.12 µM GSK761. The cells were then kept unstimulated (iDCs) or stimulated (maturation) with 100 ng/mL LPS for 24 h (mDCs). Differentiation and maturation markers were assessed by FACS. (**A**) Data are illustrated as FACS plots or (**B**) percentage of CD11c^+^, CD14^+^, CD80^+^, CD83^+^, CD86^+^ and CD209^+^ cells from total alive cells, *n* = 3 (**C**) by histogram or (**D**) by mean fluorescence intensity (MFI) of CD11c, CD14, CD80, CD83, CD86 and CD209 proteins, *n* = 3. ** *p* < 0.01, *** *p* < 0.001， **** *p* < 0.0001.

Given the observed effect of GSK761 in reducing some key genes required for activation, we further assessed the possible role of SP140 in regulating DC phenotype. GSK761 or a vehicle was added during the differentiation of monocytes to iDCs. After washing cells to remove GSK761, iDCs were subsequently matured (mDCs) with LPS for 24 h or kept without maturation (no LPS) (Figure 2A). We first investigated whether GSK761 affects the surface markers CD209 and CD11c that define the differentiation of monocytes into iDCs. FACS analysis showed no changes in the frequency of CD209^+^ and CD11c^+^ in vehicles compared to GSK761-treated cells (Figure 3A,B).

Protein expression levels of CD209 and CD11c were slightly downregulated and upregulated, respectively, in GSK761 pretreated cells (Figure 3C), but these differences were not statistically significant (Figure 3D). We next examined the effect of GSK761 on LPS-induced DC maturation. Interestingly, GSK761 strongly reduced the frequency of mDCs expressing the maturation marker CD83 (Figure 3A,B), as well as the levels of CD83 surface protein expression (Figure 3C,D). As expected, the co-stimulatory molecules CD80 and CD86 molecules were highly expressed on the surface of mDCs compared to iDCs. However, pretreatment with GSK761 strongly diminished the LPS-induced surface protein expression of CD80 and CD86 (Figure 3C,D) but not the frequency of CD80^+^ and CD86^+^ cells (Figure 3A,B).

**Figure 4:**
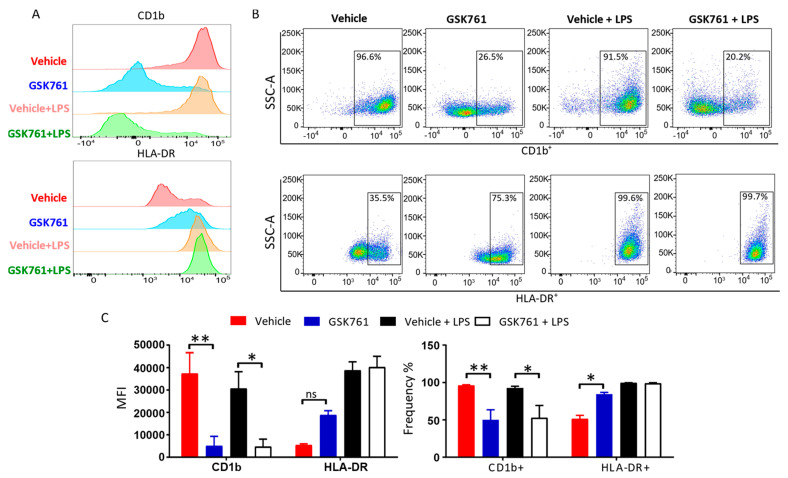
GSK761 impairs the surface expression of CD1b protein. Surface molecules involved in antigen presentation; CD1b and HLA-DR were assessed by FACS. Data are illustrated as (**A**) histogram, (**B**) FACS plots or (**C**) by MFI (**left**) or percentage of positive cells from total alive cells (**right**), *n* = 3. Statistical significance is indicated as follow: * *p* < 0.05, ** *p* < 0.01.

We then investigated whether SP140 plays a role in controlling MHC expression. CD1b, which is involved in the presentation of lipid antigens to T cells [27], was strongly reduced by GSK761 on the surface of both iDCs and mDCs (Figure 4A,C), as was the frequency of CD1b^+^ cells (Figure 4B,C). DCs maturation is normally accompanied by the redistribution of MHC class II molecules such as HLA-DR from the lysosomal MHCII compartment to the membrane to mediate the presentation of peptide antigens [28], and enhanced surface expression of HLA-DR was observed in control mDCs (Figure 4A–C). Interestingly, GSK761 increased the expression of membrane HLA-DR in iDCs (Figure 4A–C). However, there was no change in membrane HLA-DR protein expression in mDCs (Figure 4A–C).

### 2.4. GSK761-Treatment of DCs Decreases Revaxis-Induced TBX21 and Increases FOXP3 Expression in Autologous T Cells

**Figure 5:**
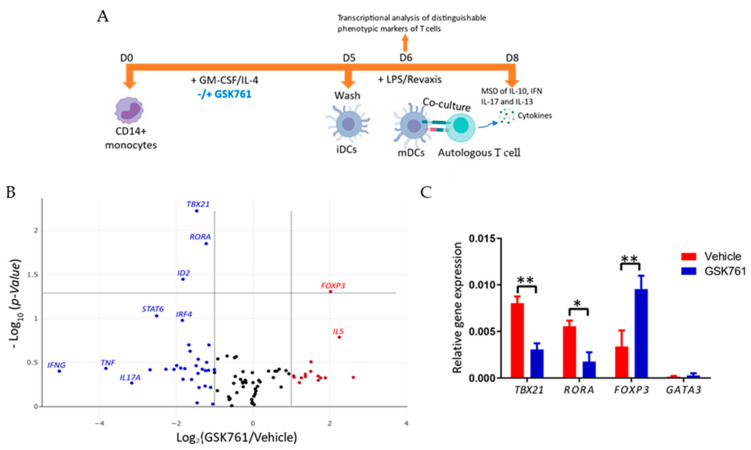
GSK761 enhances the tolerogenic potential of DCs. (**A**) Human primary CD14^+^ monocytes were incubated with 30 ng/mL GM-CSF and 20 ng/mL IL-4 for 5 days in presence of vehicle (0.1% DMSO) or 0.12 µM GSK761. iDCs were washed with PBS and co-cultured with autologous T cells (ratio DCs:T cells 1:10) in presence of 100 ng/mL LPS (for DCs maturation) and Revaxis (for antigen processing and presentation by DCs). The illustration was created with BioRender.com. (**B**) RT^2^ Profiler™ PCR Array Human T Helper Cell Differentiation was performed after 24 h of co-culture on T cells purified from DC using CD209 beads, *n* = 3. (**C**) Relative gene expression of different T-cell markers; Th1 (*TBX21*), Th2 (*GATA3*), Th17 (*RORA*) and Treg (*FOXP3*), *n* = 3. Statistical significance is indicated as follow: * *p* < 0.05, ** *p* < 0.01.

**Figure 6:**
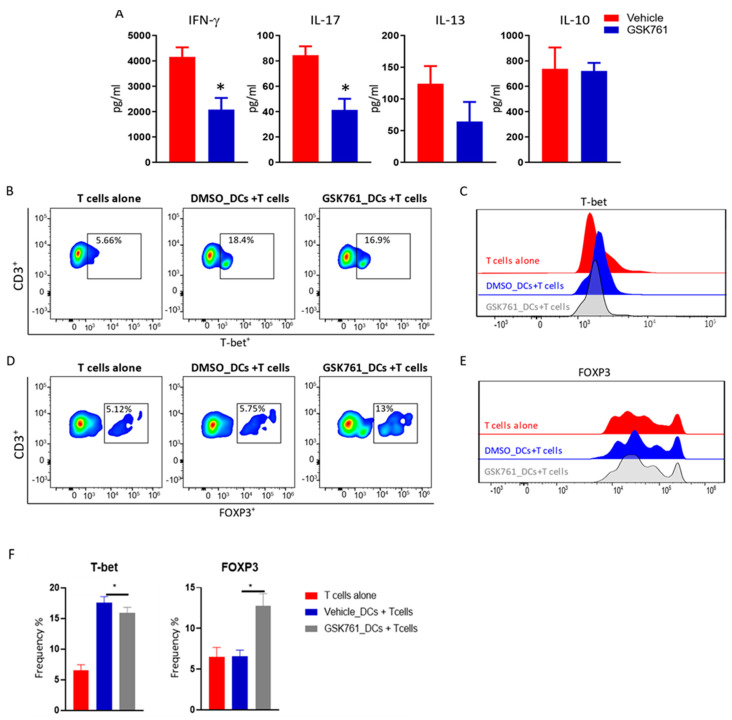
GSK761-treated DCs enhances the frequency of Foxp3^+^ T cells and reduces Th1 marker proteins. Human primary CD14^+^ monocytes were incubated with 30 ng/mL GM-CSF and 20 ng/mL IL-4 for 5 days in presence of vehicle (0.1% DMSO) or 0.12 µM GSK761. iDCs were washed with PBS and co-cultured with autologous T cells (ratio DCs:T cells 1:10) in presence of 100 ng/mL LPS (for DCs maturation) and Revaxis (for antigen processing and presentation by DCs). As control, T cells alone (without DCs) were cultured to demonstrate that adding Revaxis+LPS_DCs drives T cells to express T-bet protein. After 72 h of co-culture, the supernatant was collected for T-cells subsets specific cytokine analysis and T cells were harvested for FACS analysis of intracellular transcription marker proteins: T-bet and Foxp3. (**A**) IFN-γ, IL-17, IL-10 and IL-13 protein levels were measured by MSD in the supernatant, *n* = 3. (**B**) FACS analysis is illustrated as FACS plots for CD3^+^ and T-bet^+^ cells, (**C**) histogram for T-bet protein expression (**D**), FACS plots for CD3^+^ and Foxp3^+^ cells and (**E**) histogram for Foxp3 protein expression. (**F**) Column graphs represent the frequency of CD3^+^ T-bet^+^ cells and CD3^+^ FOXP3^+^ cells, *n* = 3. Statistical significance is indicated as follow: * *p* < 0.05.

We next investigated the effect of GSK761 on the capacity of DCs to activate and polarize autologous T cells in an antigen-specific manner. To this end, we utilized Revaxis, the 3-in-1 diphtheria, polio, and tetanus vaccine; pulsing of DCs with Revaxis allows for the stimulation of antigen-specific memory cells in vaccinated individuals [17,29]. Vehicle- or GSK761-treated iDC were incubated with LPS and Revaxis antigens and co-cultured with autologous CD4^+^ T cells (Figure 5A). It has been reported that Revaxis induces the pro-inflammatory Th1 phenotype [30,31]. Notably, the TF *Id2* previously reported to be necessary for Th1 cell generation during infection [32] was reduced in T cells co-cultured with GSK761-treated DCs (Figure 5B). In addition, GSK761 attenuated the ability of DCs to induce expression of key Th1 *TBX21* (gene encoding T-bet) [33] and Th17 *RORA* [34] lineage-defining TFs in autologous T cells while conversely enhancing their ability to induce expression of the key Treg TF *FOXP3* [35] (Figure 5B,C). Interestingly, the increase in *FOXP3* expression was associated with a decrease in *IRF1* (Supplementary Table S1), which has been reported to negatively affect Treg differentiation by repressing *FOXP3* expression [36]. Although there was no change in *GATA3* expression, the expression of Th2-associated cytokine genes such as *IL4* and *IL5* [37,38] was increased in T cells that were co-cultured with GSK761-treated DCs (Supplementary Table S1) while expression of Th1-associated *IFNγ* and *TNF* [38,39] and Th17-associated *IL17A* [40], were downregulated (Figure 5B,C and Supplementary Table S1). In accordance with the changes at the mRNA level, IFN-γ and IL-17 protein secretion by T cells was significantly reduced when co-cultured with GSK761- pretreated DCs compared to vehicle-treated DCs (Figure 6A). IL-13 (Th2 marker [38]) was unaffected (Figure 6A).

Finally, we validated the previous observations by staining the intracellular TF proteins that identify Th1 and Treg phenotype, T-bet, and FOXP3, respectively. FACS analysis showed that T cells co-cultured with GSK761-treated DCs exhibit a slight reduction in the frequency of T-bet^+^ cells (Figure 6B) but a marked decrease in T-bet protein expression (Figure 6C). GSK761 treatment clearly enhanced the potential of DCs to increase the frequency of FOXP3^+^ T cells (Figure 6D). No change in protein expression of FOXP3 amongst FOXP3^+^ cells was observed (Figure 6E). Overall, these data suggest that SP140 inhibition favors FOXP3-expressing Treg cells at the expense of Th1 and Th17 cell generation, thus enhancing the tolerogenic potential of DCs.

## 3. Discussion

Previous in vitro and in vivo studies have demonstrated the strong anti-inflammatory effects of BET Brd inhibitors that target BRD2, BRD3, BRD4, and BRDT [17,18,19]. The BET proteins regulate the expression of a plethora of genes, and due to this and their ubiquitous expression, therapeutic translation into the clinic has been limited by multiple adverse events [41,42]. SP140 is predominantly expressed in immune cells, and its expression is upregulated upon inflammation [10]. In addition, SP140 has been associated with several autoimmune and inflammatory diseases through genetic and epigenetic association studies [10,11,12], suggesting a strong rationale for therapeutic targeting of the SP140 protein. GSK761 is the first small molecule inhibitor of SP140 protein and has recently been used to demonstrate the role of SP140 in regulating the inflammatory transcriptional program in macrophages ex vivo [10]. We previously described the synthesis of GSK761 and demonstrated a high degree of affinity and specificity of this compound for SP140 protein at the concentrations used in this study [10]. Here, we investigated the capacity of GSK761 to modify DC function and its capacity to induce T-cell activation in vitro.

The aim of ChIP-qPCR experiments was to evaluate the distribution of SP140 on chromatin changes in response to LPS stimulation. Since transcription of pro-inflammatory cytokines such as *TNFα* and *IL1β* begins shortly after LPS stimulation and achieves maximal levels by 2–6 h, we focused ChIP experiments on early time points where changes in SP140 binding might be relevant to the early regulation of pro-inflammatory cytokine gene expression. This kinetic mechanism was also seen in our previous study in macrophages using the same compound [10]. In the current article, Figure 1D shows that SP140 protein is maximally enriched at the TSS of pro-inflammatory genes at relatively early time points (4–6 h) following LPS stimulation in DCs, consistent with a role in regulating the expression of these genes. The enrichment of SP140 at those genes was extremely low after 24 h of LPS, comparable to baseline (pre-LPS level). Since peak cellular SP140 protein levels are apparent at much later time points, the implication is that the factors required for SP140 recruitment to these sites (e.g., chromatin modifications, other recruited proteins) are no longer present at that later time point and that SP140 plays a different role at those times; the specific mechanisms will need to be explored in future studies.

In autoimmune and inflammatory diseases such as RA and CD, DCs play a central role in contributing to disease pathology by producing inflammatory cytokines and expressing co-stimulatory molecules, which promote the activation of inflammatory T cells [16,43,44]. In this study, SP140 was shown to be critical for both the in vitro maturation of DCs and their response to inflammatory stimuli. In line with our previous study in macrophages [10], we found that in DCs, LPS induced an increase in SP140 expression and recruitment of the protein to the TSS of pro-inflammatory cytokines. Further, GSK761 inhibited the expression of DC-induced cytokines required for inflammatory T-cell activation, including IL12-p70, TNF, IL-1β, and IL-6. In addition, GSK761 impaired DC maturation, which was indicated by a strong reduction in the expression of maturation surface markers CD83 and the co-stimulatory molecules CD80 and CD86. CD80 and CD86 molecules work in tandem to provide co-stimulatory signals necessary for T-cell activation and survival [45].

While the mechanism by which SP140 regulates co-stimulatory molecules remains to be determined in DCs, our recent study in human macrophages showed *CD80* and *CD83* as SP140 protein-bound genes [10]. GSK761 inhibited SP140 binding to those genes and decreased their expression [10]. The TF STAT1 is crucial for DCs maturation and acts by controlling the expression of co-stimulatory molecules [25]. In this study, GSK761 reduced *STAT1* gene expression, providing a possible mechanism for the inhibitory effects of the compound on DC maturation. iDCs exhibit high expression of the chemokine receptors CCR1 and CCR5, which enables migration to areas of inflammation, while mDCs lose cell surface expression of CCR1 and CCR5 [26]. We found that following treatment with GSK761, DCs retain high expression levels of *CCR1* and *CCR5,* further indicating that SP140 inhibition hampered LPS-induced maturation and kept DCs in an immature state. During the steady state, DCs maintain T-cell tolerance through multiple mechanisms, including inducing anergy, deletion, and Treg activity [46]. In addition, SP140 was found to be critical in controlling CD83 protein expression in DCs. CD83 was reported to stabilize MHC-II on their surface, permitting positive CD4^+^ T-cell selection [47]. Recombinant CD83 proteins and anti-CD83 antibodies that exploit the function of CD83 were demonstrated to be effective in the treatment of various mouse models of autoimmune and inflammatory diseases (such as graft rejection, RA, and inflammatory bowel disease) [48,49,50,51,52].

Autoimmune diseases are associated with an excessive activation and proliferation of inflammatory T-cells such as Th1 cells [53]. The therapeutic efficacy of inhibiting DC:T-cell interactions in multiple autoimmune diseases has already been shown using Abatacept which binds to CD80/86 on DCs, preventing the co-stimulatory signal required for T-cell activation [54,55]. Considering this, we sought to investigate the effect of GSK761 on DCs in this respect. To allow appropriate measurement of the effect of GSK761, we used an assay to prime human DCs and assess T-cell activation using Revaxis (inactivated diphtheria, −tetanus, and −poliomyelitis vaccine) as described in [17,56]. Revaxis is used in this study as a means to stimulate an antigen-specific T-cell response, which we feel is the most relevant function of DCs. Because the frequency of T cells specific for any given antigen in ‘naïve’ humans is extremely low, studying antigen-specific human T-cell responses is challenging. However, the use of Revaxis overcomes this challenge since most people have been vaccinated against the components (diphtheria, tetanus, polio) of this vaccine and hence harbor expanded populations of memory T cells responsive to these antigens. Utilizing Revaxis-treated DCs has been shown to effectively stimulate human recall responses, including T cells polarized towards Th1 and Th17 phenotypes [30,31,56]. GSK761 attenuated the capacity of Revaxis-treated DCs to induce *TBX21* and *RORA* expression in T cells (Th1 and Th17 phenotypes, respectively) while enhancing *FOXP3* expression (Treg phenotype). In addition, the production of cytokines that mark Th1 and Th17, such as IFN-γ [33] and IL-17 [40], respectively, were dampened, while some key TFs mediating Th1/Treg balance, such as *Id2* and *IRF1*, were strongly reduced in T-cells co-cultured with GSK761-DCs, further indicating that SP140 inhibition in DCs promotes T-cell polarization into a Treg phenotype. Id2 was shown to promote Th1 development [32], and IRF1 was reported to suppress Treg differentiation [36]. These data were supported by the observations that GSK761-treated DCs induced a higher percentage of FOXP3^+^ T cells while lowering T-bet expression in T-bet^+^ T cells.

We propose that SP140 inhibition in DCs may induce Tregs by virtue of their inability to mature and to express the required cytokines and co-stimulatory molecules, in agreement with earlier reports that iDCs induce higher numbers of Tregs [57]. Additionally, the enhanced Treg generation could be linked to an impaired capacity to uptake and process or present the antigen by DCs. Interestingly, although HLA-DR expression was not affected, GSK761 inhibited the surface expression of CD1b in both iDCs and mDCs. CD1b has been suggested to be implicated in MS [58] and infections [59]. Since the CD1b protein has been described as a surface molecule responsible for the presentation of both foreign and self-lipid antigens to T-cells [27], it is a relevant and important contributor to DC effector function. Altogether, this study indicates the role of SP140 in the regulation of antigen presentation in DCs, as well as therapeutic potential in this respect.

While GSK761 has been shown to strongly and selectively inhibit the SP140 protein and reduce the inflammatory response in DCs (in vitro) and macrophages (in vitro and ex vivo) [10], GSK761 is not suitable for evaluating the effects of SP140 inhibition in in vivo animal models due to the poor pharmacokinetic properties of this molecule as discussed in [10]. As discussed above, by extensively characterizing the properties of GSK761, we established this molecule to have the critical attributes that make it suitable for use as an in vitro/ex vivo tool to probe SP140 function in human cells. GSK761 may help to elucidate the role of SP140 as a gene regulator in a manner that would not be possible through, for example, knockout or knock-down studies that simply remove the protein. This has allowed us to significantly extend our understanding of SP140 function and highlight its potential as a therapeutic target.

This study suggests that in DCs, GSK761 treatment affects certain cytokines more significantly at 24 h (TNF and IL-10) and others at 4 h (IL1β, IL-6, IFNγ, and IL8). In a previous study [10], we observed variable binding kinetics of SP140 to different gene sets at different time points after LPS stimulation. This included SP140 binding to the CCL5 TSS at 1 h and to the CCL2 TSS only after 4 h [10]. The factors responsible for these differences in binding kinetics are currently unknown and may include differences in the chromatin environment and the kinetics of transcription factor activation and binding. Therefore, the observed differences in the duration of the effect of GSK761 on cytokine production could be attributed to the differential kinetics of SP140 binding to gene regulatory regions.

There are several limitations to our study. Firstly, because Revaxis was included in all of these experiments, we are currently unable to conclude whether GSK761-modified DCs would impact the CD4^+^ T-cell response similarly in the context of alternative antigenic stimuli or even in an antigen-independent manner. A second limitation of our study is that measuring cytokine secretion in the supernatant may not fully capture the complexity of the T-cell response over time and can also be confounded by cytokine secretion from DCs in the co-culture. An alternative method, such as the measurement of intracellular cytokine staining by flow cytometry, could provide more detailed information concerning the major cellular sources of the cytokines. A third limitation is that we currently do not know to what extent changes in total cytokine production reflect a reduced expansion of responding cells vs direct modulation of cytokine production. Future studies could investigate whether GSK761-treated DCs induced less T-cell proliferation using Ki67 [60] as a marker. It would also be valuable to explore whether antigen-specific cells only convert or expand after contact with GSK761-treated DCs to understand the mechanism underlying the observed conversion to Foxp3-expressing regulatory T cells.

In conclusion, this study identifies SP140 as an interesting target for the modulation of DC function, with the potential for generating tolerogenic DCs. The therapeutic rationale of SP140 targeting in DCs, particularly in in vivo models of autoimmune and inflammatory diseases, remains to be confirmed in future studies after developing a new in vivo-suitable SP140 inhibitor compound.

## 4. Materials and Methods

The human biological samples were sourced ethically, and their research use was in accord with the terms of the informed consent under an IRB/EC-approved protocol. Written informed consent was obtained from each donor, as approved by the UK East of England—Cambridgeshire and Hertfordshire Research Ethics Committee.

### 4.1. CD14^+^ Monocyte Isolation, In Vitro Generation of DCs and SP140 Inhibition

Peripheral blood mononuclear cells (PBMC) were isolated from whole blood of healthy donors (supplied from the GSK Stevenage Blood Donation Unit and Sanquin Institute Amsterdam, Amsterdam, The Netherlands (Project number B07.002-X)) using Ficoll density centrifugation (Fisher Scientific, Waltham, MA, USA). CD14^+^ monocytes were isolated from PBMCs using a positive selection kit (Miltenyi Biotech, Leiden, The Netherlands) according to the manufacturer’s protocol.

To examine the expression of SP140 in DCs; CD14^+^ monocytes were incubated for 5 days with GM-CSF (R&D Systems, Abingdon, UK; 30 ng/mL) and IL-4 (R&D Systems; 20 ng/mL) to generate immature monocytes derived dendritic cells (iDCs) as described in [17,20,21,22,23,61]. This protocol is well established to generate immature DC-like in terms of phenotype, functions, and surface markers (such as the high expression of CD11c, CD1b, and CD209 and the very low expression of CD14 and CD83) [20,21,22,23,24]. iDC were left unstimulated or were stimulated with LPS (Salmonella Typh, Sigma, Zwijndrecht, The Netherlands; 100 ng/mL) for 4 h to assess the *SP140* gene expression or for 4, 6, and 24 h to assess SP140 binding to chromatin.

To investigate the possible role of SP140 in mediating DC differentiation, maturation, and activation; CD14^+^ monocytes were incubated for 5 days with GM-CSF (R&D Systems; 30 ng/mL) and IL-4 (R&D Systems; 20 ng/mL) in presence of 0.1% DMSO control (Vehicle) or 0.12 µM of SP140 inhibitor compound (GSK761). iDCs were left without stimulation (naïve) or were stimulated with 100 ng/mL LPS (maturation) for 4 or 24 h. After 4 h the cells were harvested for transcriptional analysis. After 24 h, iDCs and mature DCs (mDCs) were harvested for FACS analysis of differentiation and maturation surface markers and co-stimulatory molecules. The supernatant was harvested after 4 and 24 h of LPS stimulation for cytokine production analysis.

In all experiments, cells were cultured in RPMI 1640 medium (Lonza, Geleen, The Netherlands) supplemented with 10% FCS, 2 mM l-glutamine (Lonza), 100 units/mL penicillin (Lonza), and 100 μg/mL streptomycin (Lonza).

### 4.2. siRNA-Mediated SP140 Knockdown

iDCs (generated as described above) were transfected with siGENOME human smartpool SP140 siRNA or non-targeting scrambled siRNA with DharmaFECT™ transfection reagents according to manufacturer’s protocol (Dharmacon). The cells were then stimulated with 100 ng/mL LPS for 4 h (for qPCR) or 24 h (for ELISA). The supernatant was harvested for cytokine measurement, and the cells were lysed (ISOLATE II RNA Lysis Buffer RLY-Bioline, London, UK) for RNA extraction.

### 4.3. Isolation of Human Autologous CD4+ T Cells and DCs-T Cells Co-Culture

After CD14^+^ positive selection (as described above), human autologous CD4^+^ T cells were isolated from eluted PBMCs using a negative selection kit (Miltenyi Biotech, Leiden, The Netherlands) according to the manufacturer’s protocol. CD4^+^ T cells were incubated for 5 days in RPMI 1640 medium supplemented with 10% FCS, 2 mM L-glutamine, 100 units/mL penicillin, and 100 μg/mL streptomycin containing IL-7 (R&D Systems; 10 ng/mL). Vehicle- or GSK761-pretreated iDCs (as described above) were co-cultured with autologous T cells (ratio of 1:10) in presence of vaccine antigens in Revaxis (Sanofi Pasteur MSD, Cambridge, MA, USA, PA54413511) (dilution 1:50) and 100 ng/mL LPS (to induce maturation and to activate the inflammatory response). After one day of co-culture, T cells were purified from DCs using CD209 (DC-SIGN) MicroBead Kit (Miltenyi Biotech, Leiden, The Netherlands) according to the manufacturer’s protocol. The purified T cells were lysed and stored at −80 °C for RNA isolation and transcriptional analysis. After 72 h of co-culture, the supernatant was collected for cytokine analysis of T-cell subsets, and T cells were harvested for flow cytometry (FACS) analysis of intracellular transcription marker proteins; T-bet and FOXP3.

### 4.4. Chromatin Immunoprecipitation (ChIP)

iDCs were generated in vitro from human primary CD14^+^ monocytes, as described above. iDCs were either left unstimulated or stimulated with 100 ng/mL LPS for 4, 6, and 24 h. The cells were cross-linked with 1% formaldehyde (Sigma) for 10 min at RT and quenched with 2.5 M glycine (Sigma) for 5 min at RT. The ChIP assay was performed using the Millipore iDeal ChIP kit for Transcription Factors (Diagenode, Denville, USA), and sonication was performed using the Picoruptor^TM^ (Diagenode, Denville, USA) according to the manufacturer’s protocols. Chromatin shearing was verified by migration on a 1% agarose gel (E-Gel, Thermo-Fisher) and visualized using E-Gel imager (Thermo-Fisher, Swindon, UK). Immunoprecipitation was performed with a polyclonal SP140 antibody (H00011262-M07; Abnova, Niefern-Öschelbronn, Germany). DNA was purified using Zymo ChIP DNA Clean & Concentrator Kit (Cambridge Bioscience, Cambridge, UK) according to the manufacturer’s protocol. ChIP-qPCR was performed on DNA isolated from input (unprecipitated) chromatin and SP140 ChIP DNA with primer pairs specific for the TSS of *TNF*, *IL-1β*, and *IL-10* genes. IgG was used as a negative control to help differentiate non-specific background signal from specific antibody signal. PCR primer sequences are provided in Supplementary Table S2. Quantitative PCR was performed using SYBR Green (Applied Biosystems, Bleiswijk, The Netherlands) and ABI PRISM^®^ 7700 SDS machine (Applied Biosystems, Bleiswijk, The Netherlands). Results were quantitated using the delta–delta CT (ΔΔCT) method.

### 4.5. Cytotoxicity Assay

DCs were generated in vitro from human primary CD14^+^ monocytes as described above. DCs were plated into opaque-walled 96-well plate and incubated with a concentration gradient of GSK761 (0.04–1.11 μM) for 1 h (0.1% vehicle was used as control). The cells were then stimulated with 100 ng/mL LPS for 24 h. Cell viability was assessed using CellTiter-Glo^®^ Luminescent T-cell Viability Assay kit (Promega, Madison, WI, USA) according to the manufacturer’s protocol. This assay quantifies ATP, an indicator of metabolically active cells, as described in [62,63]. Briefly, an equal volume of freshly prepared CellTiter-Glo^®^ reagent was added to each well; the plate was shaken for 10 min at RT, and luminescent signals were recorded using a plate reader SpectraMax M5 (Molecular Devices, San Jose, CA, USA). The index of cellular viability was calculated as the fold change of luminescence with respect to untreated control cells.

### 4.6. T^2^ Profiler qPCR-Array and qPCR

Total RNA was isolated from DCs or T cells using RNeasy Mini Kit (Qiagen, Hilden, Germany) and treated with DNaseI (Qiagen) according to the manufacturer’s instructions. RNA was reverse transcribed using the First Strand Synthesis Kit (Qiagen) and loaded onto a Dendritic and Antigen Presenting Cell RT^2^ profiler qPCR array or RT^2^ Profiler™ PCR Array Human T Helper Cell Differentiation, according to the manufacturer’s protocol (Qiagen) and run on QuantStudio 7 Flex (software v1.0). Qiagen’s online GeneGlobe Data Analysis Center (https://geneglobe.qiagen.com/us/analyze/, accessed on 1 February 2020) was used to determine the differentially expressed genes. The data were presented as volcano plot. PCR amplification of *SP140* was performed by Fast Start DNA Master^plus^ SYBR Green I kit on the Light Cycler 480 (Roche, Applied Science, Basel, Switzerland) or on 7900HT Fast Real-Time PCR System (Applied Biosystems). All data were normalized to the geometric mean of two reference genes, *B2M* and *RPLP0*.

### 4.7. Cytokine Analysis

Cytokine levels in the supernatant of vehicle- or GSK761-pretreated DCs (stimulated with 100 ng/mL LPS for 4 or 24 h) were measured using electro-chemiluminescence assays (Meso Scale Discovery [MSD])-Human ProInflammatory 7-Plex Tissue Culture Kit (IFN-γ, IL-1β, IL-6, IL-8, IL-10, IL-12p70, TNF) according to manufacturer’s protocols. Secreted cytokine levels of TNFα, IL1-β from SP140 siRNA or scrambled treated DCs were quantified in collected supernatant using the DuoSet^®^ ELISA Development Systems according to the manufacturer’s protocol (R&D systems™). The samples were analyzed on an MSD 1250 Sector Imager 2400 (Mesoscale, Rockville, MD, USA) or Synergy HT Multi-Detection Microplate Reader (BioTek, Winooski, VT, USA) for MSD or ELISA, respectively.

### 4.8. Flow Cytometry (FACS)

Further, 2 × 10^5^ cells were plated into 96 well round/vbottom plates and subsequently washed with PBS, and then incubated with Live/dead stain (diluted 1:1000) for 20 min at RT (in the dark). Cells were then washed in FACS buffer followed by incubation with 50 µL Fc Block for 15 min at RT. Antibody cocktail was made up according to Supplementary Tables S3 and S4. A total of 100 µL of stain was added to the 50 µL cells/block solution and incubated for a further 20–30 min at 4 °C. As controls for all stains, fluorescence minus one (FMO) was generated and incubated with cells as above in order to determine correct gating for cell populations. For intracellular staining of transcription factors, a permeabilization step was performed using 1% Saponin (Sigma-Aldrich). All sample data were acquired using the BD FACSCanto II Flow Cytometer with FACS Diva software version 8.01 (BD BioSciences, Franklin Lakes, NJ, USA) and analyzed using FlowJo v10.

### 4.9. Statistics Analysis

Statistical analysis was performed with GraphPad Prism v8.0.2.263 (GraphPad Software Inc.). For group analysis, data were subjected to one-way ANOVA or Student’s *t*-test. The two-tailed level of significance was set at *p* ≤ 0.05 (*), 0.01 (**), 0.001 (***) or 0.0001 (****) for group differences. Data are shown as mean ± SEM. The figures were prepared using Inkscape 0.92.4.

## Data Availability

Not applicable.

## Supplementary Materials

The following are available online at https://www.mdpi.com/article/10.3390/cimb45050269/s1, Supplementary Figure S1: Validation of the in vitro generation of DCs; Supplementary Figure S2: LPS stimulation does not induce SP140 recruitment to *IL10-TSS*; Supplementary Figure S3: At ≤0.12 µM, GSK761 showed no cytotoxicity; Supplementary Figure S4: Treatment with *SP140* siRNA reduces pro-inflammatory cytokines secretion by DCs; Supplemental Table S1: GSK671 decreases the capacity of DCs to induce inflammatory T-cell phenotypes; Supplemental Table S2: Oligonucleotide primers; Supplemental Table S3: Antibodies for surface protein staining; Supplemental Table S4: Antibodies for CD3 and intracellular proteins staining.

## References

[1] Lewis K.L., Reizis B. (2012). Dendritic Cells: Arbiters of Immunity and Immunological Tolerance. Cold Spring Harb. Perspect. Biol..

[2] Merad M., Manz M.G. (2009). Dendritic cell homeostasis. Blood.

[3] Wardowska A., Komorniczak M., Bullo-Piontecka B., Dȩbska-Ślizień M.A., Pikuła M. (2019). Transcriptomic and Epigenetic Alterations in Dendritic Cells Correspond with Chronic Kidney Disease in Lupus Nephritis. Front. Immunol..

[4] Nencioni A., Beck J., Werth D., Grünebach F., Patrone F., Ballestrero A., Brossart P. (2007). Histone deacetylase inhibitors affect dendritic cell differentiation and immunogenicity. Clin. Cancer Res..

[5] Brogdon J.L., Xu Y., Szabo S.J., An S., Buxton F., Cohen D., Huang Q. (2006). Histone deacetylase activities are required for innate immune cell control of Th1 but not Th2 effector cell function. Blood.

[6] Ge Z., Da Y., Xue Z., Zhang K., Zhuang H., Peng M., Li Y., Li W., Simard A., Hao J. (2013). Vorinostat, a histone deacetylase inhibitor, suppresses dendritic cell function and ameliorates experimental autoimmune encephalomyelitis. Exp. Neurol..

[7] Woo S.J., Lee S.-M., Lim H.S., Hah Y.-S., Jung I.D., Park Y.-M., Kim H.-O., Cheon Y.-H., Jeon M.-G., Jang K.Y. (2016). Myeloid deletion of SIRT1 suppresses collagen-induced arthritis in mice by modulating dendritic cell maturation. Exp. Mol. Med..

[8] Elmesmari A., Alivernini S., Tolusso B., Bui L., Vaughan D., Gigante M., Federico F., Ferraccioli G., Gremese E., McInnes I. (2018). SAT0003 Synovial tissue cd1c+ dendritic cells in rheumatoid arthritis express high levels of the epigenetic regulator of inflammation, microrna-155 and inflammatory cytokines. Ann. Rheum. Dis..

[9] Fraschilla I., Jeffrey K.L. (2020). The Speckled Protein (SP) Family: Immunity’s Chromatin Readers. Trends Immunol..

[10] Ghiboub M., Koster J., Craggs P.D., Yim A.Y.F.L., Shillings A., Hutchinson S., Bingham R.P., Gatfield K., Hageman I.L., Yao G. (2022). Modulation of macrophage inflammatory function through selective inhibition of the epigenetic reader protein SP140. BMC Biol..

[11] Matesanz F., Potenciano V., Fedetz M., Ramos-Mozo P., Abad-Grau M.D.M., Karaky M., Barrionuevo C., Izquierdo G., Ruiz-Peña J.L., García-Sánchez M.I. (2015). A functional variant that affects exon-skipping and protein expression of *SP140* as genetic mechanism predisposing to multiple sclerosis. Hum. Mol. Genet..

[12] Mehta S., Cronkite D.A., Basavappa M., Saunders T.L., Adiliaghdam F., Amatullah H., Morrison S.A., Pagan J.D., Anthony R.M., Tonnerre P. (2017). Maintenance of macrophage transcriptional programs and intestinal homeostasis by epigenetic reader SP140. Sci. Immunol..

[13] Zucchelli C., Tamburri S., Filosa G., Ghitti M., Quilici G., Bachi A., Musco G. (2018). Sp140 is a multi-SUMO-1 target and its PHD finger promotes SUMOylation of the adjacent Bromodomain. Biochim. Biophys. Acta Gen. Subj..

[14] Li J., Zhao G., Gao X. (2013). Development of neurodevelopmental disorders: A regulatory mechanism involving bromodomain-containing proteins. J. Neurodev. Disord..

[15] Ghiboub M., Elfiky A.M.I., de Winther M.P.J., Harker N.R., Tough D.F., de Jonge W.J. (2021). Selective Targeting of Epigenetic Readers and Histone Deacetylases in Autoimmune and Inflammatory Diseases: Recent Advances and Future Perspectives. J. Pers. Med..

[16] Coutant F., Miossec P. (2016). Altered dendritic cell functions in autoimmune diseases: Distinct and overlapping profiles. Nat. Rev. Rheumatol..

[17] Schilderink R., Bell M., Reginato E., Patten C., Rioja I., Hilbers F.W., Kabala P.A., Reedquist K.A., Tough D.F., Tak P.P. (2016). BET bromodomain inhibition reduces maturation and enhances tolerogenic properties of human and mouse dendritic cells. Mol. Immunol..

[18] Toniolo P.A., Liu S., Yeh J.E., Moraes-Vieira P.M., Walker S.R., Vafaizadeh V., Barbuto J.A.M., Frank D.A. (2015). Inhibiting STAT5 by the BET Bromodomain Inhibitor JQ1 Disrupts Human Dendritic Cell Maturation. J. Immunol..

[19] Sun Y., Wang Y., Toubai T., Oravecz-Wilson K., Liu C., Mathewson N., Wu J., Rossi C., Cummings E., Wu D. (2015). BET bromodomain inhibition suppresses graft-versus-host disease after allogeneic bone marrow transplantation in mice. Blood.

[20] Sallusto F., Lanzavecchia A. (1994). Efficient presentation of soluble antigen by cultured human dendritic cells is maintained by granulocyte/macrophage colony-stimulating factor plus interleukin 4 and downregulated by tumor necrosis factor alpha. J. Exp. Med..

[21] Romani N., Gruner S., Brang D., Kämpgen E., Lenz A., Trockenbacher B., Konwalinka G., Fritsch P.O., Steinman R.M., Schuler G. (1994). Proliferating dendritic cell progenitors in human blood. J. Exp. Med..

[22] Hiasa M., Abe M., Nakano A., Oda A., Amou H., Kido S., Takeuchi K., Kagawa K., Yata K., Hashimoto T. (2009). GM-CSF and IL-4 induce dendritic cell differentiation and disrupt osteoclastogenesis through M-CSF receptor shedding by up-regulation of TNF-α converting enzyme (TACE). Blood.

[23] Chometon T.Q., Siqueira M.D.S., Sant’anna J.C., Almeida M.R., Gandini M., Nogueira A.C.M.D.A., Antas P.R.Z. (2020). A protocol for rapid monocyte isolation and generation of singular human monocyte-derived dendritic cells. PLoS ONE.

[24] Cao W., Lee S.H., Lu J. (2004). CD83 is preformed inside monocytes, macrophages and dendritic cells, but it is only stably expressed on activated dendritic cells. Biochem. J..

[25] Jackson S.H., Yu C.-R., Mahdi R.M., Ebong S., Egwuagu C.E. (2004). Dendritic Cell Maturation Requires STAT1 and Is under Feedback Regulation by Suppressors of Cytokine Signaling. J. Immunol..

[26] Sallusto F., Schaerli P., Loetscher P., Schaniel C., Lenig D., Mackay C.R., Qin S., Lanzavecchia A. (1998). Rapid and coordinated switch in chemokine receptor expression during dendritic cell maturation. Eur. J. Immunol..

[27] Shahine A. (2018). The intricacies of self-lipid antigen presentation by CD1b. Mol. Immunol..

[28] ten Broeke T., Wubbolts R., Stoorvogel W. (2013). MHC class II antigen presentation by dendritic cells regulated through endosomal sorting. Cold Spring Harb. Perspect. Biol..

[29] Gajdos V., Soubeyrand B., Vidor E., Richard P., Boyer J., Sadorge C., Fiquet A. (2011). Immunogenicity and safety of combined adsorbed low-dose diphtheria, tetanus and inactivated poliovirus vaccine (REVAXIS (^®®^)) versus combined diphtheria, tetanus and inactivated poliovirus vaccine (DT Polio (^®®^)) given as a booster dose at 6 years of age. Hum. Vaccin..

[30] Rybczynska M., Baudry J., Klaus E. (2020). The impact of frost-damage on the quality and quantity of the secreted antigen-specific IgG repertoire. Vaccine.

[31] Roberts C.A., Durham L.E., Fleskens V., Evans H.G., Taams L.S. (2017). TNF Blockade Maintains an IL-10+ Phenotype in Human Effector CD4+ and CD8+ T Cells. Front. Immunol..

[32] Shaw L.A., Bélanger S., Omilusik K.D., Cho S., Scott-Browne J.P., Nance J.P., Goulding J., Lasorella A., Lu L.-F., Crotty S. (2016). Id2 reinforces TH1 differentiation and inhibits E2A to repress TFH differentiation. Nat. Immunol..

[33] Kanhere A., Hertweck A., Bhatia U., Gökmen M.R., Perucha E., Jackson I., Lord G.M., Jenner R.G. (2012). T-bet and GATA3 orchestrate Th1 and Th2 differentiation through lineage-specific targeting of distal regulatory elements. Nat. Commun..

[34] Unutmaz D. (2009). RORC2: The master of human Th17 cell programming. Eur. J. Immunol..

[35] Li Z., Li D., Tsun A., Li B. (2015). FOXP3+ regulatory T cells and their functional regulation. Cell Mol. Immunol..

[36] Fragale A., Gabriele L., Stellacci E., Borghi P., Perrotti E., Ilari R., Lanciotti A., Remoli A.L., Venditti M., Belardelli F. (2008). IFN Regulatory Factor-1 Negatively Regulates CD4+CD25+ Regulatory T Cell Differentiation by Repressing Foxp3 Expression. J. Immunol..

[37] Hartenstein B., Teurich S., Hess J., Schenkel J., Schorpp-Kistner M., Angel P. (2002). Th2 cell-specific cytokine expression and allergen-induced airway inflammation depend on JunB. EMBO J..

[38] O’garra A., Arai N. (2000). The molecular basis of T helper 1 and T helper 2 cell differentiation. Trends Cell Biol..

[39] Britt R.D., Thompson M.A., Sasse S.K., Pabelick C.M., Gerber A.N., Prakash Y.S. (2019). Th1 cytokines TNF-α and IFN-γ promote corticosteroid resistance in developing human airway smooth muscle. Am. J. Physiol. Cell Mol. Physiol..

[40] Annunziato F., Cosmi L., Santarlasci V., Maggi L., Liotta F., Mazzinghi B., Parente E., Filì L., Ferri S., Frosali F. (2007). Phenotypic and functional features of human Th17 cells. J. Exp. Med..

[41] Alqahtani A., Choucair K., Ashraf M., Hammouda D.M., Alloghbi A., Khan T., Senzer N., Nemunaitis J. (2019). Bromodomain and extra-terminal motif inhibitors: A review of preclinical and clinical advances in cancer therapy. Future Sci. OA.

[42] Andrieu G., Belkina A.C., Denis G.V. (2016). Clinical trials for BET inhibitors run ahead of the science. Drug Discov. Today Technol..

[43] Wehr P., Purvis H., Law S., Thomas R. (2018). Dendritic cells, T cells and their interaction in rheumatoid arthritis. Clin. Exp. Immunol..

[44] Knight S.C. (2016). Dendritic Cell-T-Cell Circuitry in Health and Changes in Inflammatory Bowel Disease and Its Treatment. Dig. Dis..

[45] Chen L., Flies D.B. (2013). Molecular mechanisms of T cell co-stimulation and co-inhibition. Nat. Rev. Immunol..

[46] Vendelova E., Ashour D., Blank P., Erhard F., Saliba A.-E., Kalinke U., Lutz M.B. (2018). Tolerogenic Transcriptional Signatures of Steady-State and Pathogen-Induced Dendritic Cells. Front. Immunol..

[47] Tze L.E., Horikawa K., Domaschenz H., Howard D.R., Roots C.M., Rigby R., Way D.A., Ohmura-Hoshino M., Ishido S., Andoniou C. (2011). CD83 increases MHC II and CD86 on dendritic cells by opposing IL-10–driven MARCH1-mediated ubiquitination and degradation. J. Exp. Med..

[48] Xu J.-F., Huang B.-J., Yin H., Xiong P., Feng W., Xu Y., Fang M., Zheng F., Wang C.-Y., Gong F.-L. (2006). A limited course of soluble CD83 delays acute cellular rejection of MHC-mismatched mouse skin allografts. Transpl. Int..

[49] Lan Z., Ge W., Arp J., Jiang J., Liu W., Gordon D., Healey D., Debenedette M., Nicolette C., Garcia B. (2010). Induction of Kidney Allograft Tolerance by Soluble CD83 Associated with Prevalence of Tolerogenic Dendritic Cells and Indoleamine 2,3-Dioxygenase. Transplantation.

[50] Eckhardt J., Kreiser S., Döbbeler M., Nicolette C., DeBenedette M.A., Tcherepanova I.Y., Ostalecki C., Pommer A.J., Becker C., Günther C. (2014). Soluble CD83 ameliorates experimental colitis in mice. Mucosal. Immunol..

[51] Royzman D., Andreev D., Stich L., Rauh M., Baeuerle T., Ellmann S., Boon L., Kindermann M., Peckert K., Bozec A. (2019). Soluble CD83 Triggers Resolution of Arthritis and Sustained Inflammation Control in IDO Dependent Manner. Front. Immunol..

[52] Li Z., Ju X., Silveira P.A., Abadir E., Hsu W.-H., Hart D.N.J., Clark G. (2019). CD83: Activation Marker for Antigen Presenting Cells and Its Therapeutic Potential. Front. Immunol..

[53] Skapenko A., Leipe J., Lipsky P.E., Schulze-Koops H. (2005). The role of the T cell in autoimmune inflammation. Arthritis Res. Ther..

[54] Korhonen R., Moilanen E. (2009). Abatacept, a novel CD80/86-CD28 T cell co-stimulation modulator, in the treatment of rheumatoid arthritis. Basic Clin. Pharmacol. Toxicol..

[55] Noisette A., Hochberg M.C. (2018). Abatacept for the treatment of adults with psoriatic arthritis: Patient selection and perspectives. Psoriasis Targets Ther..

[56] De Vries L.C., Ghiboub M., van Hamersveld P.H., Welting O., Verseijden C., Bell M.J., Rioja I., Prinjha R.K., Koelink P.J., Strobl B. (2021). Tyrosine kinase 2 signalling drives pathogenic T-cells in colitis. J. Crohn’s Colitis.

[57] Lutz M.B., Schuler G. (2002). Immature, semi-mature and fully mature dendritic cells: Which signals induce tolerance or immunity?. Trends Immunol..

[58] Bine S., Haziot A., Malikova I., Pelletier J., Charron D., Boucraut J., Mooney N., Gelin C. (2012). Alteration of CD1 expression in multiple sclerosis. Clin. Exp. Immunol..

[59] Olivier M., Foret B., Le Vern Y., Kerboeuf D., Guilloteau L.A. (2013). Plasticity of Migrating CD1b+ and CD1b- Lymph Dendritic Cells in the Promotion of Th1, Th2 and Th17 in Response to Salmonella and Helminth Secretions. PLoS ONE.

[60] Di Rosa F., Cossarizza A., Hayday A.C. (2021). To Ki or Not to Ki: Re-Evaluating the Use and Potentials of Ki-67 for T Cell Analysis. Front. Immunol..

[61] Huang Y., Min S., Lui Y., Sun J., Su X., Liu Y., Zhang Y., Han D., Che Y., Zhao C. (2012). Global mapping of H3K4me3 and H3K27me3 reveals chromatin state-based regulation of human monocyte-derived dendritic cells in different environments. Genes Immun..

[62] Węsierska-Gądek J., Gueorguieva M., Ranftler C., Zerza-Schnitzhofer G. (2005). A new multiplex assay allowing simultaneous detection of the inhibition of cell proliferation and induction of cell death. J. Cell Biochem..

[63] Zhelev Z., Ohba H., Bakalova R., Hadjimitova V., Ishikawa M., Shinohara Y., Baba Y. (2004). Phenothiazines suppress proliferation and induce apoptosis in cultured leukemic cells without any influence on the viability of normal lymphocytes. Cancer Chemother. Pharmacol..

