# Supplementary material for: The Epigenetic Reader Protein SP140 Regulates Dendritic Cell Activation, Maturation and Tolerogenic Potential
Source: Curr Issues Mol Biol. 2023 May 11;45(5):4228–45. doi: 10.3390/cimb45050269 (PMC10217447; doi:10.3390/cimb45050269)
Supplement: Supplementary file 1 [file cimb-45-00269-s001.zip › cimb-2377740-supplementary.pdf]

current issues in molecular biology

Supplemental Figures

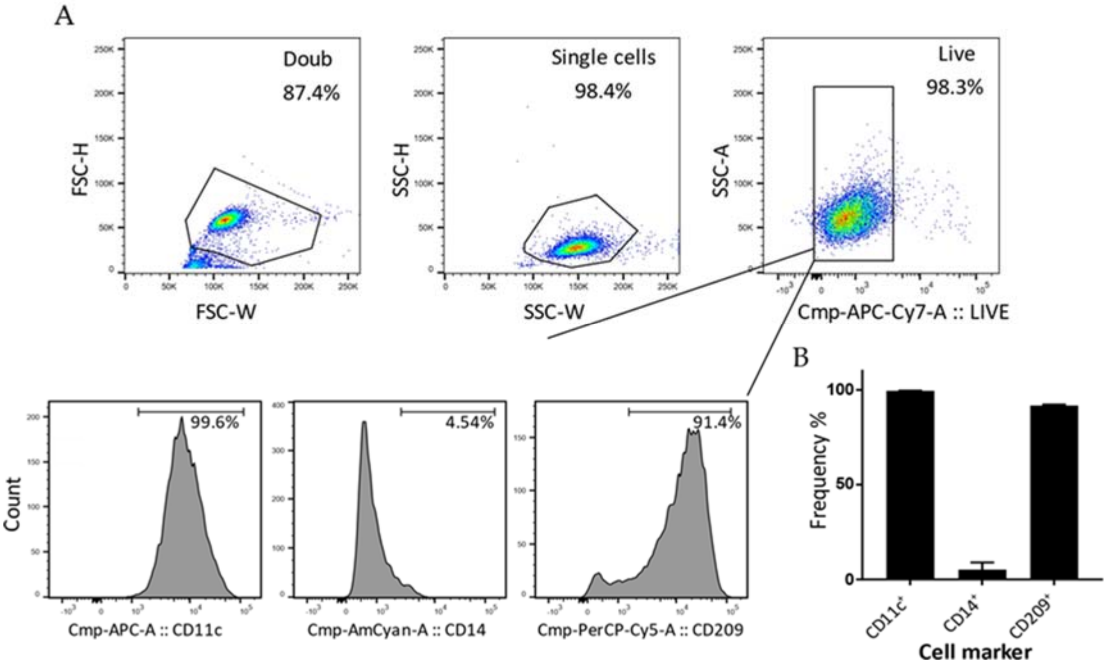

Supplementary Figure S1: Validation of the in vitro generation of DCs. Human primary CD14+ monocytes were incubated with 30 ng/mL GM-CSF and 20 ng/mL IL-4 for 5 days. **(A)** Gating strategy to define the successful differentiation of monocytes into iDCs based on CD11c and CD209 surface markers and to select the alive cells. **(B)** Frequency of CD11c+, CD209+ (DCs marker) and CD14+ (monocyte marker) cells from total alive cells, n=3.

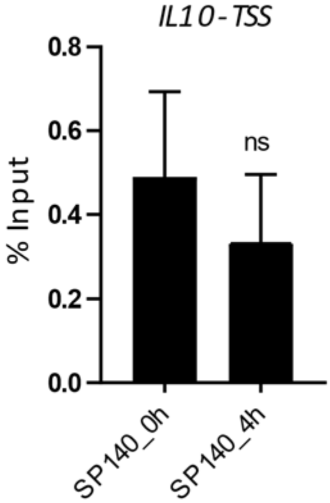

Supplementary Figure S2: LPS stimulation does not induce SP140 recruitment to *IL10-TSS*. ChIP-qPCR of SP140 protein occupancy at TSS of *IL10* (n=3) in naïve iDCs or after 4 hours of 100 ng/mL LPS-stimulation

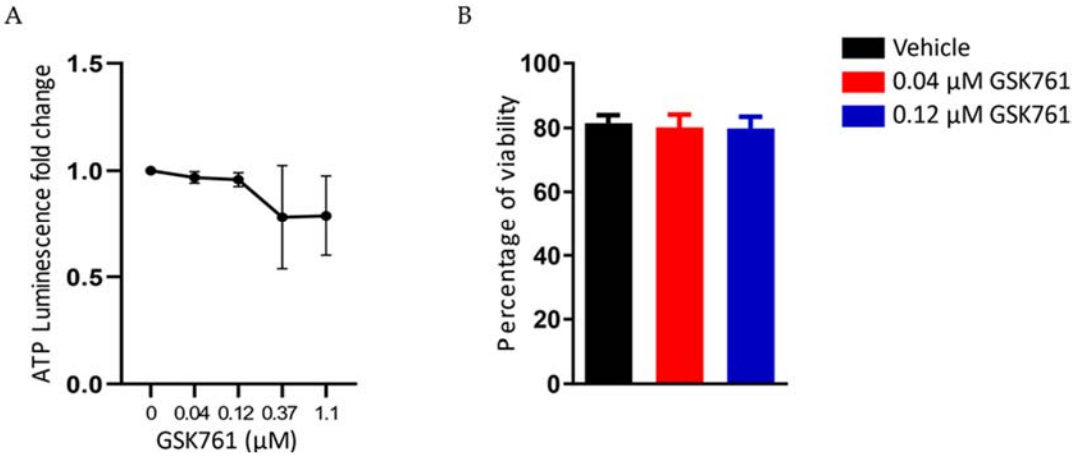

Supplementary Figure S3: At ≤0.12 μM, GSK761 showed no cytotoxicity. (A) ATP luminescence measurement within DCs treated with an increasing concentration of GSK761 (0.01, 0.04, 0.12, 0.37. 1.11 μM) or 1% vehicle (DMSO) in presence of 100 ng/mL of LPS for 24h, n=4. (**B**) Live/dead cells were analyzed by FACS of Vehicle- or 0.04 or 0.12 μM GSK761 treated-DCs and stimulated with 100 ng/mL LPS for 24 hours, n=3.

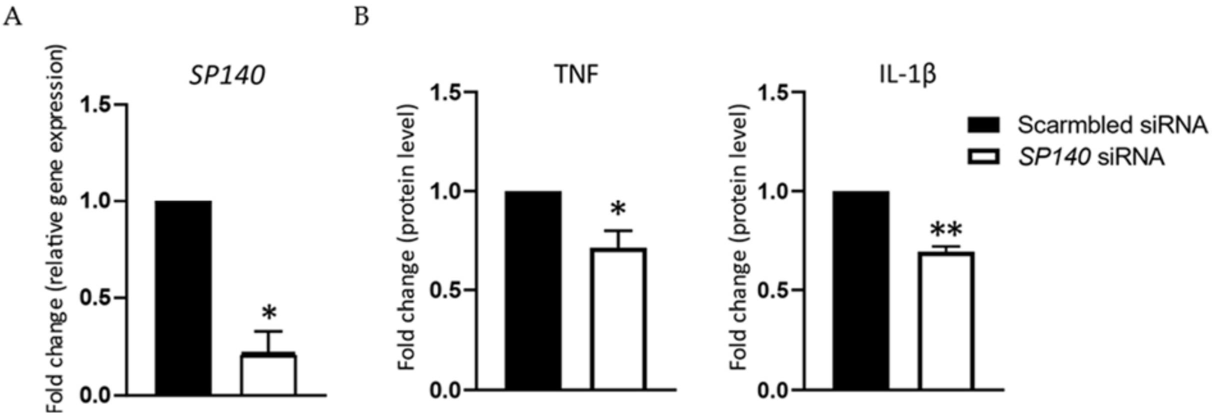

Supplementary Figure S4: Treatment with *SP140* siRNA reduces pro-inflammatory cytokines secretion by DCs. iDCs (generated as described previously) were transfected with *SP140* siRNA or non-targeting scrambled siRNA. The cells were then stimulated with 100 ng/mL LPS for **(A)** 4 hours to measure the gene expression of SP140 by qPCR (n=3) or **(B)** for 24 hours to measure protein levels of TNF and IL-1β by ELISA, n=3. Statistical significance is indicated as follow: *$P < 0.05$, **$P < 0.01$.

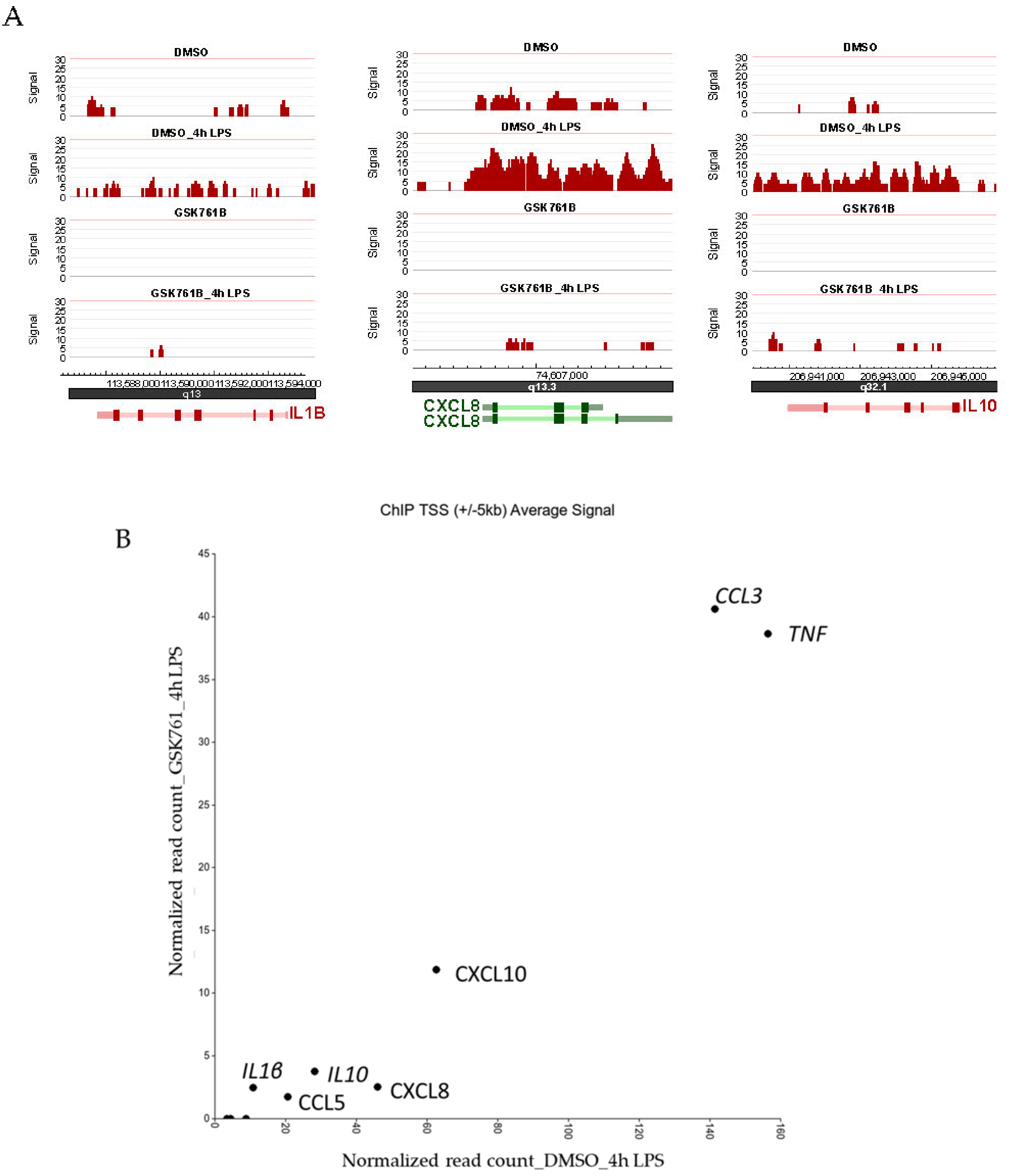
A
DMSO
DMSO_4h LPS
GSK761B
GSK761B_4h LPS
Signal
IL1B
CXCL8
CXCL8
IL10
q13
q13.3
q32.1
B
ChIP TSS (+/-5kb) Average Signal
CCL3
TNF
CXCL10
IL1β
IL10
CCL5
CXCL8
Normalized read count_GSK761_4h LPS
Normalized read count_DMSO_4h LPS

Supplementary Figure S5: Treatment with GSK761 reduces the binding of SP140 protein to TSS of pro-inflammatory cytokine genes. (A) SP140 ChIP-seq genome browser view of *IL1β, CXCL8* and *IL10*. Y axis represents a signal score of recovered sequences in unstimulated or LPS-stimulated (100 ng/mL for 4 hours) macrophages that were pretreated with 0.1% DMSO or 0.04 µM GSK761. **(B)** TSS plot comparing some of differentially SP140-bound cytokine/chemokine genes after 4h of 100 ng/mL LPS stimulation of 0.1% DMSO- and 0.04 µM GSK761-treated inflammatory macrophages. The figures were retrieved from publicly available SP140 ChIP-seq data that have been deposited at the European Genome-phenome Archive: EGAS00001004460.

Supplementary Tables

Genes	Fold down-regulation
IFNG	-33.01
TNF	-14.22
IL17A	-8.93
NR4A3	-6.41
STAT6	-5.69
REL	-4.81
TNFRSF9	-4.16
TGIF1	-3.97
IRF8	-3.68
IRF4	-3.58
ID2	-3.53
STAT1	-3.53
CCR5	-3.18
IRF1	-3.11
CEBPB	-3.03
RUNX1	-2.79
TBX21	-2.76
ASB2	-2.74
IL13RA1	-2.7
IL4R	-2.69
IL12B	-2.65

Genes	Fold up-regulation
PERP	6.13
IL5	4.76
FOXP3	4.07
PTGDR2	3.7
HOXA3	3.4
CCL7	3.26
GATA4	3.25
IL9	2.86
IL4	2.84
LRRC32	2.58
TNFSF11	2.58

Supplemental Table S1: GSK671 decreases the capacity of DCs to induce inflammatory T cell phenotypes. Human primary CD14$^+$ monocytes were incubated with 30 ng/mL GM-CSF and 20 ng/mL IL-4 for 5 days in presence of vehicle (0.1% DMSO) or 0.12 µM GSK761. iDCs were washed with PBS and co-cultured with autologous T cells (ratio DCs:T cells 1:10) in presence of 100 ng/mL LPS (for DCs maturation) and Revaxis (for antigen processing and presentation by DCs). RT^2 Profiler™ PCR Array Human T Helper Cell Differentiation was performed after 24 hours of co-culture on T cells purified from DC using CD209 beads, n=3. Tables represents genes with more than 2.5-fold down-regulation (left) or with more than 2.5-fold up-regulation (right) in T cells that were co-cultured with GSK761-pretreated DCs relative to T cells that were co-cultured with vehicle-pretreated DCs.

Gene	Forward (5′-3′)	Reverse (5′-3′)
TNF-TSS	GGGACATATAAAGGCAGTTGTTGG	TCCCTCTTAGCTGGTCCTCTGC
IL10-TSS	GAGAAGGAGGAGCTCTAAGCAG	TCAGGGAGGCCTCTTCATTC
IL1β-TSS	AAACAGCGAGGGAGAAACTGG	GGCTGAAGAGAATCCCAGAGC
SP140	AGGATGGTCGCAGAGATCCA	TGGCCTTGTTATTGCACTTGC

Supplemental Table S2: Oligonucleotide primers

Stain	Flour	Supplier	Vol/test (µl)
Live/dead	eFlour780	eBioscience	1:1000
Trustain FcX block	n/a	Biolegend	3.5/50 µL final
CD14	V500	BD Horizon	1.5
CD11c	APC	Biolegend	2.5
CD80	PE	Biolegend	2.5
CD86	BV421	Biolegend	2.5
CD83	AF488	Biolegend	2.5
CD209	PerCp Cy5.5	Biolegend	2.5
HLA-DR	BV510	Biolegend	3.5
CD1b	FITC	Biolegend	2.5

Supplemental Table S3: Antibodies for surface protein staining.

Stain	Flour	Supplier	Dilution
CD3	FITC	Biolegend	2.5:100
FOXP3	PE	eBioscience	2.5:100
T-bet	PE	eBioscience	2.5:100

Supplemental Table S4: Antibodies for CD3 and intracellular proteins staining
